# Coexistence of chronic myeloid leukemia and diffuse large B-cell lymphoma with antecedent chronic lymphocytic leukemia: a case report and review of the literature

**DOI:** 10.1186/s13256-018-1612-4

**Published:** 2018-03-11

**Authors:** Khadega A. Abuelgasim, Hinna Rehan, Maha Alsubaie, Nasser Al Atwi, Mohammed Al Balwi, Saeed Alshieban, Areej Almughairi

**Affiliations:** 1 Department of Oncology, King Abdulaziz Medical City, Ministry of National Guard Health Affairs, Riyadh, 1775 Saudi Arabia; 2 King Abdullah International Medical Research Center, King Saud University for Health Sciences, Riyadh, Saudi Arabia; 3 Pathology Department, King Abdulaziz Medical City, Ministry of National Guard Health Affairs, Riyadh, Saudi Arabia

**Keywords:** Chronic lymphocytic leukemia, Chronic myeloid leukemia, Richter’s transformation, Diffuse large B-cell lymphoma, Purine analogs, Tyrosine kinase inhibitors

## Abstract

**Background:**

Chronic lymphocytic leukemia and chronic myeloid leukemia are the most common types of adult leukemia. However, it is rare for the same patient to suffer from both. Richter’s transformation to diffuse large B-cell lymphoma is frequently observed in chronic lymphocytic leukemia. Purine analog therapy and the presence of trisomy 12, and *CCND1* gene rearrangement have been linked to increased risk of Richter’s transformation. The coexistence of chronic myeloid leukemia and diffuse large B-cell lymphoma in the same patient is extremely rare, with only nine reported cases. Here, we describe the first reported case of concurrent chronic myeloid leukemia and diffuse large B-cell lymphoma in a background of chronic lymphocytic leukemia.

**Case presentation:**

A 60-year-old Saudi man known to have diabetes, hypertension, and chronic active hepatitis B was diagnosed as having Rai stage II chronic lymphocytic leukemia, with trisomy 12 and rearrangement of the *CCND1* gene in December 2012. He required no therapy until January 2016 when he developed significant anemia, thrombocytopenia, and constitutional symptoms. He received six cycles of fludarabine, cyclophosphamide, and rituximab, after which he achieved complete remission.

One month later, he presented with progressive leukocytosis (mostly neutrophilia) and splenomegaly. Fluorescence *in situ* hybridization from bone marrow aspirate was positive for translocation (9;22) and reverse transcription polymerase chain reaction detected *BCR-ABL* fusion gene consistent with chronic myeloid leukemia. He had no morphologic or immunophenotypic evidence of chronic lymphocytic leukemia at the time. Imatinib, a first-line tyrosine kinase inhibitor, was started. Eight months later, a screening imaging revealed new liver lesions, which were confirmed to be diffuse large B-cell lymphoma.

**Conclusions:**

In chronic lymphocytic leukemia, progressive leukocytosis and splenomegaly caused by emerging chronic myeloid leukemia can be easily overlooked. It is unlikely that chronic myeloid leukemia arose as a result of clonal evolution secondary to fludarabine treatment given the very short interval after receiving fludarabine. It is also unlikely that imatinib contributed to the development of diffuse large B-cell lymphoma; rather, diffuse large B-cell lymphoma arose as a result of Richter’s transformation. Fludarabine, trisomy 12, and *CCND1* gene rearrangement might have increased the risk of Richter’s transformation in this patient.

## Background

Chronic lymphocytic leukemia (CLL) is the most common type of leukemia in the Western world. It is characterized by progressive accumulation of mature B or T lymphocytes in the peripheral blood, bone marrow, liver, and lymphoid organs [1, 2].

CML is a chronic myeloproliferative neoplasm with an annual incidence of 1–2 cases per 100,000 people per year. It originates from abnormal hematopoietic stem cells induced by the *BCR-ABL* oncogene, resulting in the involvement of multiple hematopoietic lineages, but predominantly myeloid cells [3].

Despite the frequent occurrence of CLL and CML in adults, it is rare for the two to coexist [4–9]. The association between lymphoproliferative disorders (LPD) and myeloid malignancies, such as acute myeloid leukemia, myelodysplastic syndrome, or myeloproliferative disorders, is not well understood; however, it is possible that two malignant clones might arise from the same cancerous stem cell [10].

In CLL, Richter’s transformation (RT) to Hodgkin’s lymphoma (HL) and non-Hodgkin’s lymphoma (NHL), including diffuse large B-cell lymphoma (DLBCL), is frequently observed and usually has a poor outcome [11, 12].

To the best of our knowledge, CLL, CML, and DLBCL have never been reported to coexist. Here we describe the first case of the coexistence of CML and DLBCL in a patient with antecedent CLL.

## Case presentation

**Fig. 1:**
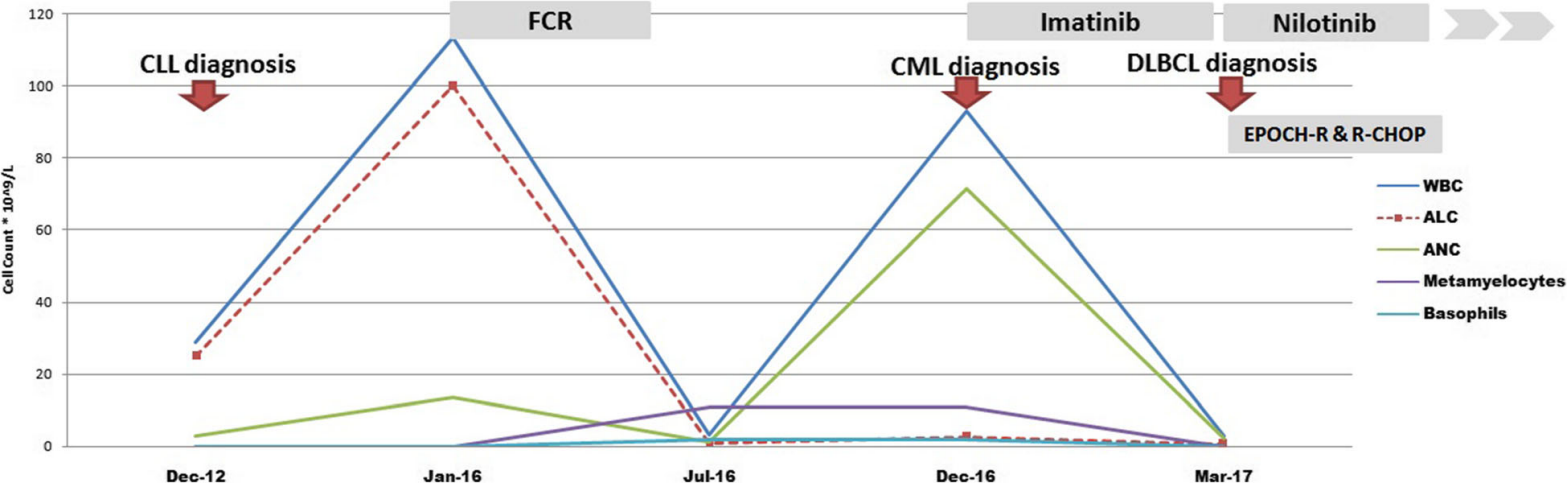
The patient was diagnosed as having chronic lymphocytic leukemia in 2012, with chronic myeloid leukemia in 2016, and with diffuse large B-cell lymphoma in 2017. ALC absolute lymphocyte count, ANC absolute neutrophil count, CLL chronic lymphocytic leukemia, CML chronic myeloid leukemia, DLBCL diffuse large B-cell lymphoma, EPOCH-R etoposide, cyclophosphamide, doxorubicin, vincristine, and rituximab; FCR fludarabine, cyclophosphamide, prednisone and rituximab; R-CHOP rituximab, cyclophosphamide, doxorubicin, oncovine (vincristine) and prednisone, WBC white blood cell count

**Fig. 2:**
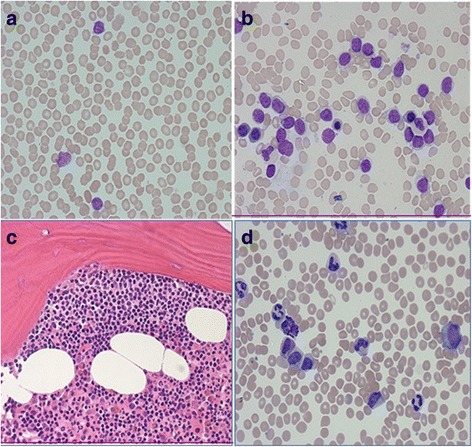
Bone marrow and peripheral blood smear in 2012 consistent with a diagnosis of chronic lymphocytic leukemia and in peripheral blood smear in 2016 consistent with a diagnosis of chronic myeloid leukemia. **a** and **b** Peripheral blood smear and bone marrow aspirate showing small mature lymphocytes with condensed nuclear chromatin (× 60 high power magnification). **c** Bone marrow biopsy showing paratrabecular nodular lymphocytic infiltrate (× 40 high power magnification). **d** Peripheral blood smear showing leukocytosis, myeloid left shift, and occasional basophils and eosinophils, which suggests a diagnosis of chronic myeloid leukemia (× 60 high power magnification)

**Fig. 3:**
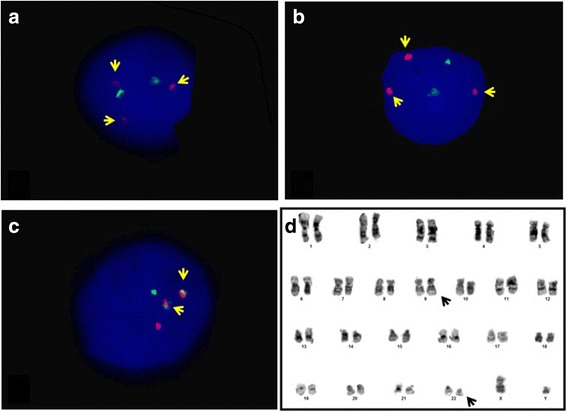
Interphase nucleus fluorescence *in situ* hybridization and cytogenetic karyotyping images from bone marrow culture at the time of chronic lymphocytic leukemia diagnosis and from peripheral blood at the time of chronic myeloid leukemia diagnosis. **a** Interphase nucleus probes with CCND1 (Red) and IGH (Green), yellow arrows indicate the presence of one large signal (normal chromosome 11) and two small signals indicate the rearrangement involving other chromosome 11 homolog. **b** Interphase nucleus probes with chromosome 8 centromere (D8Z1, Green) and chromosome 12 centromere (D12Z1, Red). Arrows indicate the presence of trisomy of chromosome 12. **c** Interphase nucleus probes for ABL1 gene (on chromosome 9, Red) and BCR gene (on chromosome 22, Green). Arrows indicate BCR/ABL fusion signals. **d** Cytogenetic karyotyping revealing a male with 46,XY, t(9;22)(q34;q11.2) translocation

A 60-year-old Saudi man with a history of diabetes, hypertension, and chronic active hepatitis B, an entrepreneur, married with seven children, who denied tobacco smoking, alcohol consumption, and illicit drug use, was initially seen at our facility and was diagnosed as having Rai stage II CLL in December 2012. His physical examination at presentation revealed a moderately built man. His respiratory and cardiovascular examination was normal. His liver was normal in size but his spleen was palpable (8 cm below the costal margin). He had generalized lymphadenopathy involving his neck, axillae, and bilateral inguinal regions; however, the lymph nodes were 1–3 cm in size. A neurological examination revealed no focal neurological deficit. His serum creatinine at presentation was 72 umol/L, and his blood urea nitrogen (BUN) was 4.3 mmol/L. The results of his liver function test were: aspartate aminotransferase (AST) 22 unit/L, alanine transaminase (ALT) 27 unit/L, total bilirubin 12.1 umol/L, albumin 3.5 g/dL, and alkaline phosphatase of 68 unit/L. His blood count at presentation showed a white blood cell (WBC) count of 28.9 × 109/L, hemoglobin (Hb) level of 13.4 g/dl, a platelet count of 106 × 109/L, and an absolute lymphocyte count (ALC) of 25.1 × 109/L (Fig. 1). Peripheral blood flow cytometry revealed 43% of total acquired events co-expressing CD5, CD19, CD23, CD79b, and cytoplasmic CD79a, but lacking surface immunoglobulin light chains, CD10, and CD38. Bone marrow aspirate and biopsy (BMAB) showed hypercellular bone marrow with diffuse intestinal and focal paratrabecular lymphocytic infiltrate. The lymphocytes were mature, small, and positive for CD20, CD79a, PAX-5, CD5, CD23, and BCL2, but negative for cyclin D1 and CD10 (Fig. 2). Conventional cytogenetic tests and fluorescence *in situ* hybridization (FISH) revealed that 27% of analyzed cells displayed rearrangement of the *CCND1* gene in chromosome 11 and 15% of cells had trisomy 12, but t(11;14) (q13;q32) was not detected (Fig. 3).

**Fig. 4:**
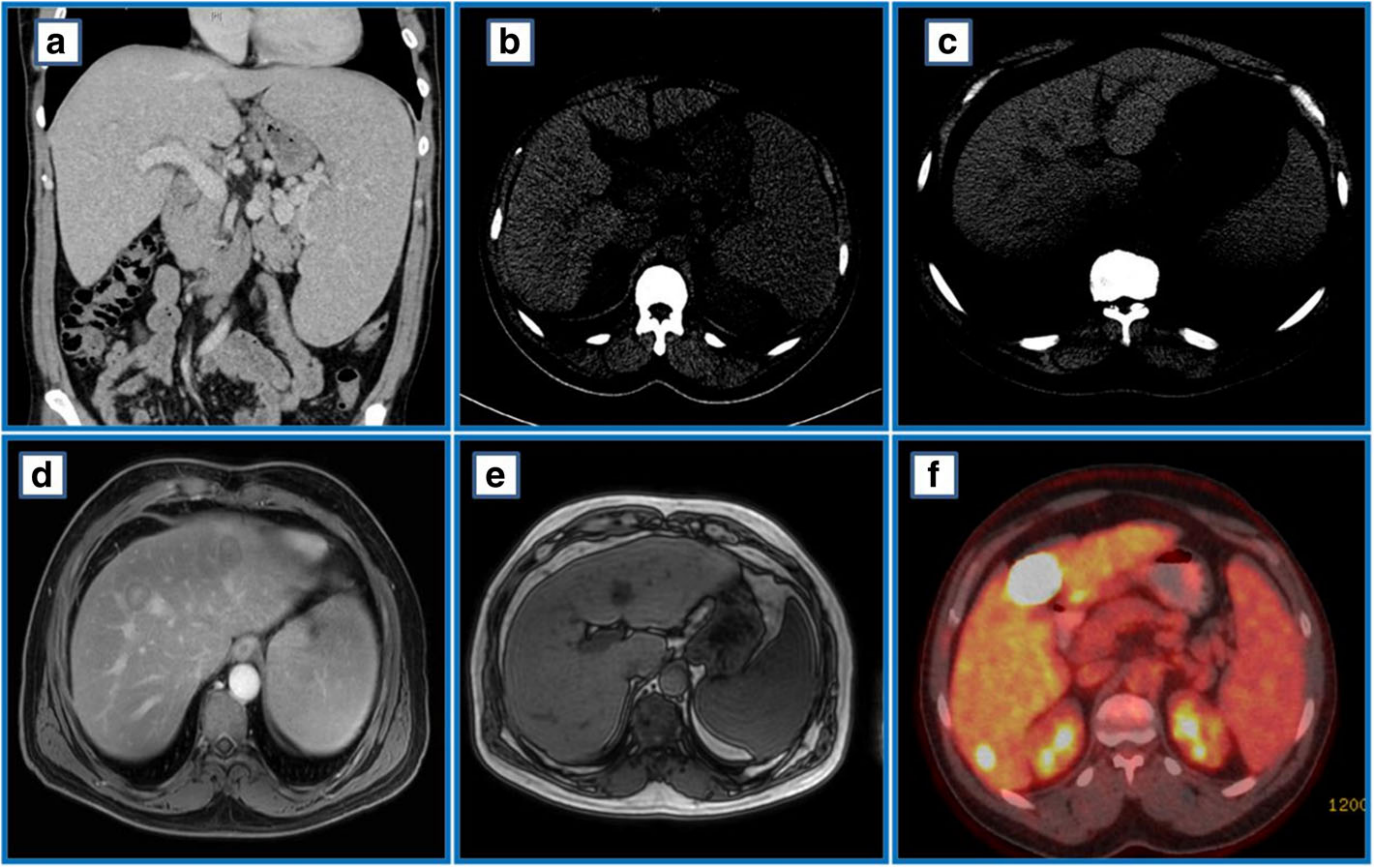
Serial imaging at the time of chronic lymphocytic leukemia diagnosis and at the time of diffuse large B-cell lymphoma diagnosis. **a** and **b** Abdominal computed tomography scan in July 2012 showing hepatosplenomegaly with hypodense liver lesions. **c** Abdominal computed tomography in March 2017 showing hepatomegaly with hypodense liver lesions but no splenomegaly. **d** and **e** Abdominal magnetic resonance imaging in April 2017 showing multiple liver lesions appearing slightly high in T2 and low in T1, with restriction in diffusion-weighted images. **f** Abdominal positron emission tomography/computed tomography in June 2017 showing multiple hypermetabolic liver lesions

**Fig. 5:**
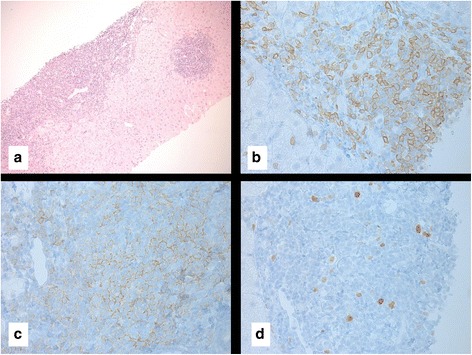
Liver biopsy taken in 2012 consistent with a diagnosis of chronic lymphocytic leukemia. There is diffuse infiltration of portal tracts by small lymphoid cells with extension to lobules and sinuses, (**a**) × 200. The lymphoid cells express CD5 (**b**) and CD23 immunostains (**c**). They show low proliferation rate (< 5%) by Ki-67 immunostain (**d**)

A computed tomography (CT) scan of his neck, chest, abdomen, and pelvis showed hepatomegaly with focal hypodense liver lesions and massive splenomegaly (20.9 × 7.3 cm) (Fig. 4a, b). A liver biopsy showed infiltration with small lymphocytic lymphoma (SLL; Fig. 5). He remained under surveillance until January 2016, when he developed anemia, thrombocytopenia, and significant constitutional symptoms. He was started on chemoimmunotherapy with fludarabine 25 mg/m^2^ on days 1–3, cyclophosphamide 250 mg/m^2^ on days 1–3, and rituximab 375 mg/m^2^ on day 1 (FCR) for a total of six cycles, until 27 July 2016. His blood counts normalized (Fig. 1). And an end-of-therapy CT scan showed resolution of the liver lesions, and significantly reduced lymphadenopathy and splenomegaly.

One month after completing FCR therapy, his leukocytosis (mostly neutrophilia) returned, with myeloid left shift. His spleen was palpable 10 cm below the costal margin. His WBC count reached 93 × 109/L, Hb 10.9 g/dl, platelet count 61 × 109/l, absolute neutrophil count 42 × 109/L, and eosinophil count 3 × 109/L. Peripheral blood blasts and basophils were 1% each (Fig. 1). BMAB was suboptimal. Conventional cytogenetic analysis using bone marrow showed Philadelphia chromosomes in all examined cells with no other cytogenetic aberrations. FISH analysis revealed presence of the *BCR-ABL* fusion gene in 100% of cells examined, and quantitative reverse transcription polymerase chain reaction (RT-PCR) of peripheral blood showed a ratio of *BCR-ABL/ABL* of 21% (Fig. 3).

Since the morphologic findings of both the blood smear and the cytogenetics were compatible with chronic phase CML, he was started on first-generation tyrosine kinase inhibitor (TKI) imatinib at a dose of 400 mg daily. As a result, his complete blood count normalized and his spleen shrank. Although he achieved an appropriate hematologic response within 3 months of imatinib treatment, on 21 March 2017 he demonstrated a suboptimal molecular response with a *BCR-ABL/ABL* ratio of 17%. Subsequently, he was switched to 300 mg orally administered nilotinib (a second-generation TKI) twice daily. After 3 months of nilotinib treatment, the *BCR-ABL/ABL* ratio dropped to 0.093%.

**Fig. 6:**
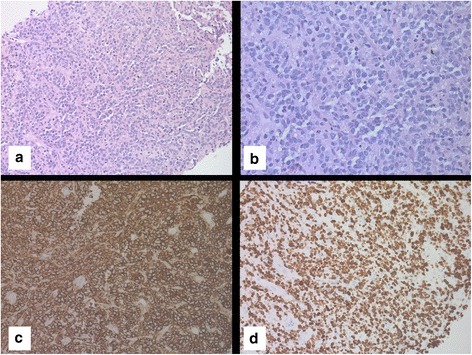
Liver biopsy taken in 2017 consistent with a diagnosis of diffuse large B-cell lymphoma. There is diffuse infiltration by large atypical lymphoid cells with increased apoptosis, × 200 (**a**) and, × 400 (**b**). The tumor cells express CD20 immunostain (**c**). They show high proliferation rate (70%) by Ki-67 immunostain (**d**)

To screen for hepatocellular carcinoma, an abdominal CT scan was done in March 2017 and it revealed multiple hypoechoic liver lesions (Fig. 4c). Magnetic resonance imaging of his liver confirmed the presence of at least nine hepatic lesions (Fig. 4d, e). Positron emission tomography (PET) showed multiple metabolically active liver lesions (Fig. 4f). A liver biopsy performed in May 2017 confirmed RT to high-grade B cell lymphoma. After negative tests for c-MYC, the final pathological diagnosis was confirmed as DLBCL (Fig. 6). Staging BMAB was morphologically negative for CLL, CML, and DLBCL. FISH analysis on BMAB was negative for *CCND1* gene rearrangement, trisomy 12, and *BCR-ABL* oncogene.

While awaiting c-MYC *in situ* hybridization he received two cycles of chemoimmunotherapy by infusion consisting of rituximab 375 mg/m^2^ on day 1, etoposide 50 mg/m^2^ on days 1–4, prednisone 60 mg/m^2^ on days 1–5, doxorubicin 10 mg/m^2^ on days 1-4, Oncovin (vincristine) 0.5 mg/m^2^ on days 1–4, and cyclophosphamide (R-EPOCH). He was then switched to rituximab 375 mg/m^2^ on day 1, cyclophosphamide 750 mg/m^2^ on day 1, doxorubicin 50 mg/m^2^ on day 1, oncovin (vincristine) 50 mg/m^2^ on day 1, vincristine 1.4 mg/m^2^ on day 1, and prednisone 100 mg days 1–5 (R-CHOP); he completed four cycles. Radiological assessment with PET/CT at the end of chemotherapy was consistent with complete metabolic response.

A donor had been identified in preparation for allogeneic hematopoietic stem cell transplantation, in case our patient’s CML proved resistant to second-line TKI treatment. He was last seen in January 2018, 8 months after his diagnosis with DLBCL with no evidence of lymphoma and his *BCR-ABL/ABL* ratio dropped to 0.01%.

## Discussion

There have been only 32 reported cases of CLL coexisting with CML in the same patient. The most commonly occurring pattern is for long-standing CLL to precede CML [4–9].

The patient in the present case report remained under careful observation for 3 years after being diagnosed as having CLL. Soon after completing fludarabine-based chemotherapy, he was diagnosed with CML. It is possible that this CML already existed before and was only detected after his CLL was brought under control. Alternatively, it is possible that CML arose as a result of clonal evolution secondary to fludarabine treatment. However, this theory is less likely given the very short interval between receiving purine analog (PNA) and CML emergence.

Despite increasing rates of complete remission in CLL associated with fludarabine-based regimens, RT still occurs, warranting continued surveillance regardless of disease status. RT to NHL, HL, and multiple myeloma develops in approximately 3%, 0.5%, and 0.1% of patients, respectively. Risk factors for RT include advanced Rai stage at diagnosis, TP53 disruption, c-MYC abnormalities, non-deletion 13q cytogenetics, CD38 gene polymorphism, and unmutated immunoglobulin heavy chain variable region gene status [10–13].

Patients treated for CLL with PNA and alkylating agents tend to have an up to three-fold increased risk of RT compared to those who have not received PNA [14, 15]. The median time from first treatment of CLL to the development of second LPD is 2.4 years [16]. Although our patient was in complete remission from CLL/SLL when he was diagnosed as having DLBCL, we believe that RT was the cause rather than *de novo* DLBCL. This is based on biopsy evidence of CLL infiltration of his liver at the time of his initial diagnosis in 2012.

The immune-compromised state of CLL patients means that they have a frequency of secondary tumors. These are not limited to LPD, but also include solid tumors such as cancers of the lung, colorectal region, bladder, breast, central nervous system, stomach, ovaries, head, neck, and testicles, as well as melanoma, sarcoma, and myeloid leukemias [17].

Cytogenetic analysis of our patient revealed trisomy 12, which is a cytogenetic abnormality frequently seen in CLL and associated with a higher frequency of RT to DLBCL attributed to activation of the NOTCH1 pathway [18]. In our patient, rearrangement of the *CCND1* gene was also observed, which might have increased his risk of RT [19].

The development of TKIs, a major breakthrough in the therapy of CML, has dramatically improved the survival of patients with CML. However, the use of TKIs may be associated with the development of secondary malignancies. Around 7% of patients with TKI-treated CML develop non-hematologic secondary malignancies. On the other hand, this increased risk may be linked to CML itself rather than to TKI treatment [20]. The coexistence of CML with DLBCL in the same patient is even rarer, with only nine cases reported to date [21–25]. In the case reported here, it is unlikely that TKI therapy contributed to the development of DLBCL; rather, DLBCL arose as a result of RT of the antecedent CLL.

## Conclusions

In CLL, progressive leukocytosis and splenomegaly caused by emerging CML might be overlooked unless care is taken to examine the white cell differential, and appropriate further tests are carried out. It is unlikely that CML arose as a result of clonal evolution secondary to fludarabine treatment given the very short interval between receiving fludarabine and CML emergence. It is also unlikely that CML therapy contributed to the development of DLBCL; rather, DLBCL arose as a result of RT of CLL. Fludarabine therapy, trisomy 12, and *CCND1* gene rearrangement might have increased the risk of RT in this patient.

To the best of our knowledge, this is the first reported case of concurrent CML and DLBCL against a background of CLL. This case represents a tremendous medical challenge, especially with the failure of CML to respond to first-line TKI and in a background of chronic liver disease.

## Abbreviations

ALC: Absolute lymphocyte count
ALT: Alanine transaminase
AST: Aspartate aminotransferase
BMAB: Bone marrow aspirate and biopsy
BUN: Blood urea nitrogen
CLL: Chronic lymphocytic leukemia
CML: Chronic myeloid leukemia
CT: Computed tomography
DLBCL: Diffuse large B-cell lymphoma
FCR: Fludarabine, cyclophosphamide and rituximab
FISH: Fluorescence in situ hybridization
Hb: Hemoglobin
HL: Hodgkin’s lymphoma
LPD: Lymphoproliferative disorders
NHL: Non-Hodgkin’s lymphoma
PET: Positron emission tomography
PNA: Purine analog
R-CHOP: Rituximab, cyclophosphamide, doxurubicin, oncovin (vincristine) and prednisone
R-EPOCH: Rituximab, etoposide, prednisone, doxorubicin, oncovin and cyclophosphamide
RT: Richter’s transformation
RT-PCR: Reverse transcription polymerase chain reaction
SLL: Small lymphocytic lymphoma
TKI: Tyrosine kinase inhibitor
WBC: White blood cell
